# The Role of Vertebral Augmentation Procedures in the Management of Multiple Myeloma

**DOI:** 10.46989/001c.92984

**Published:** 2024-02-20

**Authors:** Nishanth Thalambedu, Mudassar Kamran, Samer Al-Hadidi

**Affiliations:** 1 Hematology and Oncology University of Arkansas for Medical Sciences; 2 Interventional Radiology University of Arkansas for Medical Sciences; 3 Myeloma Center University of Arkansas for Medical Sciences

**Keywords:** Multiple myeloma, myeloma bone disease, vertebral compression fractures, vertebral augmentation procedures, kyphoplasty, vertebroplasty

## Abstract

Approximately 90% of patients with multiple myeloma experience significant pain from osseous involvement during their lifetime. Untreated osseous involvement results in vertebral compression fractures, leading to negative consequences for quality of life. Vertebral augmentation procedures, including percutaneous vertebroplasty and kyphoplasty, offer better and faster pain control and likely lower morbidity compared with non-operative interventions. Our review provides an up-to-date summary of the indications, contraindications, timing, outcomes, and potential complications of vertebral augmentation procedures to guide practicing oncologists in effectively managing bone disease in patients with multiple myeloma.

## Introduction

Multiple myeloma (MM), the second most common hematologic malignancy, is characterized by the malignant proliferation of clonal plasma cells in the bone marrow, leading to lytic bone lesions that may result in significant pain, pathological fractures and potential neurological compromise.^1–3^ Up to 90% of patients with MM develop myeloma bone disease (MBD) during their lifetime, which is defined as the involvement of myeloma in the bones, manifesting as osteolytic bone disease or osteopenia, most commonly in the spine, and leading to substantial morbidity.^4–7^

Complex interactions between myeloma cells and the vertebral bone marrow microenvironment lead to bone loss and destruction, causing a disturbance in the natural skeletal architecture and predisposing patients to vertebral compression fractures (VCFs).^8^ The most common site of VCFs in MM is the thoracic spine, followed by the lumbar and cervical spines. VCFs are known to occur at the onset of diagnosis in 34% to 64% of patients.^9^ Each VCF leads to abnormal sagittal balance and consequent altered anterior spine compressive forces, increasing the risk of further VCFs. Patients assume a kyphotic position to compensate for pain, which aggravates pain from altered facet joint mechanics. Moreover, prolonged kyphotic deformities cause pulmonary and gastric dysfunction, leading to increased morbidity, mortality, and poor quality of life (QoL).^10,11^

With advancements in understanding the biology of MM and the addition of novel therapeutics, the median survival of patients has increased in the last decade, justifying the need to focus on better interventions to improve QoL, with optimal pain control to promote good physical functioning.^12,13^ Current non-operative interventions to treat VCFs include oral and parenteral analgesics, steroids, bisphosphonates, spinal braces, and radiotherapy.^14^ While traditional non-operative management may improve pain control, it does not stabilize the VCF or minimize the progressive kyphotic deformity, which can only be achieved by vertebral augmentation procedures (VAPs), including percutaneous vertebroplasty (PVP) and percutaneous kyphoplasty (PKP). These minimally invasive outpatient procedures performed under moderate sedation provide improved and expedited pain control, thereby preventing prolonged immobilizations.^11,15,16^

In 2019, the International Myeloma Working Group (IMWG) published a consensus statement to update the recommendations for VAPs in MM (outlined in Table 1). Briefly, any patient known to have MM who presents with back pain should undergo a careful assessment to determine the severity of pain and any accompanying neurological findings. MRI should confirm this, specifically on T1-weighted and short-T1 inversion recovery sequences, to rule out any spinal cord compression and facet joint-related pain. A CT scan must be performed for spinal instability neoplastic score classification to determine spinal instability and posterior vertebral wall defects. VAPs are also considered in patients with non-healing chronic fractures with a fracture cleft on imaging, and in patients with persistent symptoms from VCFs initially treated with conservative measures for 8-12 weeks, such as external supportive devices, including thoracolumbar spinal orthosis and thermoplastic braces. Lastly, MM disease status and hematological abnormalities, particularly thrombocytopenia, should be checked to assess the optimal timing for the performance of the procedure.^2^

**Table 1.** Indications for vertebral augmentation procedures in multiple myeloma patients with vertebral compression fractures</strong>^2^

**Absolute Indications:** Persistent, significant pain from a fractured vertebral body Persistent, significant symptoms which have not resolved with normal conservative measures after 4 weeks of treatment affecting daily activities Significant pain associated with a significant change in disability in conjunction with a new event Acute-disease patient delayed for medical reasons Selective chronic fractures
**Relative Indications:** Fracture of the thoracolumbar junction (T10–L2) that could result in a significant kyphotic deformity and, therefore, morbidity Loss of vertebral body height (progressive as evidenced by sequential erect x-rays) Posterior wall defect or destruction of a pedicle/pars, which may potentially render the affected area of the spine unstable and at risk of fracture/neurological insult
**Prophylactic Indications:** **[A]Loss of vertebral height sufficient to affect functional activities** For fracture at T10–L2 (thoracolumbar junction) consider cement augmentation; below L2 is not as significant Only if progression over time; follow up with standard x-rays every 1–3 months **[B] Risk of impending fracture** Need to take into consideration the aggressive nature of the disease and patient activity. Need for clinical trials.

In this review, we focused on the procedure and timing of VAPs among patients with MM, and on complications, contraindications, and outcomes evidence supporting the use of VAPs in these patients.

## Procedure and Timing of VAPs

VAPs were first performed in the 1980s for the treatment of vertebral hemangiomas and osteolytic tumors.^17^ VAPs involve the injection of polymethylmethacrylate (PMMA) bone cement into the vertebral body cavity.^1^ Compared with PVP, PKP uses either a balloon or a curette to create a cavity within the affected vertebral body to instill high-viscosity PMMA cement. This procedure has been shown to restore lost vertebral height (VH), correct spinal deformities, and decrease the incidence of cement leakages.^18^ Recently, smaller studies have shown the effectiveness of spine jack implants, an expandable titanium intravertebral implant that provides long-term pain relief and VH restoration in VCFs secondary to MM and other etiologies.^19,20^

Even though both PVP and PKP use biplanar fluoroscopy guidance for determining the optimal location of cement injection, cement leakage into adjacent neural and vascular spaces is observed more with PVP than with PKP. In a large majority of patients, cement leakage is clinically inconsequential; however, in extreme situations, it can potentially cause pulmonary and cerebral embolism.^21^

MBD generally affects more than one vertebral level. Although it has been advocated to augment as many involved vertebral levels as feasible, recent IMWG consensus recommends treating a maximum of 3 vertebral fractures in one PVP or PKP procedure session due to PMMA toxicity and an incrementally increased risk of respiratory dysfunction associated with cement embolization.^2^ Recent studies also show the benefit of using fenestrated pedicle screws for cement augmentation to enhance the durability of spinal constructs, particularly in cancer patients with poor bone quality.^22–24^

VCFs lead to loss of VH, resulting in an anterior shift of the spinal center of gravity, leading the patients to assume a kyphotic posture, which also compensates for weak paraspinal musculature.^25^ On a longer time frame, these compensatory mechanisms are detrimental and cause pulmonary and gastric complications leading to increased morbidity.^11^ One of the goals of VAPs is to improve or restore VH and kyphotic angle, thus achieving normal anatomical alignment of the spine and thereby improving the QoL.

The IMWG consensus statement recommends early intervention with VAPS within 4-8 weeks of diagnosis of VCFs due to the cumulative benefits of pain control, improved clinical outcomes, and enhanced QoL. This recommendation stemmed from previous studies demonstrating the said benefits.^15,26–28^ However, treatment of medical complications of MM, especially renal failure, should be prioritized over VAPs. During this period of patient stabilization, pain can be treated with non-operative interventions.

## Outcomes

Compared to non-operative management, VAPs showed a statistically significant reduction in patients’ pain levels reflected by lower opioid requirements and improved pain visual analog scale (VAS) in multiple studies. Few studies compared the outcomes between PVP and PKP (Table 2). Kose et al conducted a retrospective study among 34 patients with MM and symptomatic VCFs that underwent PKP for 22 vertebral levels in 18 patients and PVP for 28 vertebral levels in 16 patients. They observed similar benefits of pain reduction and improved QoL from both procedures at 6 weeks (*P* = 0.106) but significantly better results at 6 months (*P* = 0.024) and 1 year (*P* = 0.027) post-procedure among those who underwent PKP.^29^

**Table 2.** Studies comparing the outcomes between balloon kyphoplasty (BK) and percutaneous vertebroplasty (PVP)

Study	Outcome	PKP	PVP	Conclusion
Kose et al^29^	Mean pre- and post-operative pain score	Pre-⁠op pain: 36 6 weeks: 12.13 6 months: 8.63 1 year: 9.72	Pre-⁠op pain: 37.83 6 weeks: 15.33 6 months: 12.17 1 year: 13.47	PKP and PVP offer similar reduction in pain at 6 weeks but better results at 6 months (*P* = 0.024) and 1 year post-procedure (*P* = 0.027) among those who underwent PKP
Erdem et al^30^	Reported VAS, analgesic requirements, activity level pre- and 1-month post-procedure			Pre-op versus post-op VAS: 6.9 vs. 2.7, reduction of 4.2 points (95% confidence interval (CI) 4.0–4.5) that was significant (*P* < 0.001) Reduction of analgesic requirement by 65% 1-month post-procedure (OR = 0.35; 95% CI, 0.21–0.58) compared with pre-procedure (*P* < 0.001) Improvement in good activity score to 4.2 times (95% CI, 3.1–5.8) higher post-procedure compared to pre-procedure (*P* < 0.001) No significant differences in improvements between the type of procedure performed (kyphoplasty vs. vertebroplasty or kyphoplasty + vertebroplasty) for pain relief, decreased narcotics usage, or improvement in activity (all *P* > 0.05)
Khan et al^31^	Mean pain reduction scores	367 PKP treatments	576 PVP treatments	At 1 week: 2.8 points ± 0.4 versus 2.8 points ± 0.4 (*P* = .9) 1 week-1 year: 2.5 points ± 0.4 versus 2.5 points ± 0.5 for (*P* = 1.00) >1 year: 2.9 points ± 0.6 compared with 2.7 points ± 0.4 for kyphoplasty patients (*P* = .9)
Lamida et al^32^	Improvement of VH and KD	19 PKP Mean Pre-op VH: 1.77cm Mean pre-op KD: 9.7 Mean post-op VH: 2.18cm Mean post-op KD: 7.9	18 PVP Mean Pre-op VH: 2.13cm Mean pre-op KD: 8.4 Mean post-op VH: 2.27cm Mean post-op KD: 7.4	Statistically significant VH increase from PKP compared to PVP (0.41 cm vs. 0.14 cm; *P* = 0.006).

PKP, percutaneous kyphoplasty; PVP, percutaneous vertebroplasty; VAS, visual analog scale; VH, vertebral height; KD, kyphotic deformity

Erdem et al. performed another single-center large retrospective study of 792 patients with MM who underwent 1,072 VAPs. They reported that patients who underwent PKP or PVP had an identical reduction of back pain and analgesic requirements, and improvement in activity level at 1-month post-procedure.^30^ A pooled analysis of published case series of VAPs among patients with MM by Khan et al. revealed a similar reduction of pain scores after PVP and PKP at time intervals of <1 week, <1 year, and >1 year.^31^ Overall, irrespective of the type, VAPs are shown to play a vital role in short- and long-term pain reduction from VCFs among patients with MM.

Both PVP and PKP have been shown to improve VH and kyphotic angle in multiple studies.^33^ This improvement maintained itself for up to 3.2 years in a few studies. Lamida et al observed a significant VH increase from PKP compared to PVP (0.41 cm versus 0.14 cm *P* = 0.006) with identical analgesic effects. Although the theoretical advantage of better VH and kyphotic angle correction is evident, its translation to clinical benefit is unclear.^32^

## Complications

One of the most common complications of VAPs is cement leakage outside the confines of the vertebral body, possibly secondary to excessive injection or breach of cortical integrity. Studies show that injection of PMMA cement in amounts more than required will not lead to better pain relief or radiological outcomes, but rather increases the risk of leakage.^34^ Higher rates of leakage were more frequently encountered with an average injected cement volume of 4 mL or greater during VAPs.^35^ The cement may leak into adjacent dural, vascular, or soft tissue spaces or may embolize via the vertebral veins. Although cement extravasation does not often cause clinical symptoms, it can sometimes cause serious neurological and vascular outcomes.^36,37^

Multiple studies revealed higher cement leakage rates among PVP (30%-75%) compared to PKP (8%-33%).^38^ This is also reflected in the recent 2019 IMWG consensus statement reporting higher rates of cement extrusion in PVP than in PKP procedures.^2^ However, cement leakage has been shown to not adversely affect the pain or functional scores in either PVP or PKP.^31^

There is increased stiffness of the vertebral elements after cement augmentation, which increases the future fracture risk of adjacent vertebral levels.^39^ Historically, this risk was found to be greater after PKP (45%-75%) than PVP (0%-16%). However, those studies were primarily performed in non-malignant osteoporotic VCFs.^40^ A recent meta-analysis of published cases of VCFs among patients with MM revealed similar rates of new VCFs at untreated vertebral bodies for both PVP and PKP (7.3% versus 6.8%, *P* = 0.78).^31^

## Contraindications

Some of the contraindications to performing VAPs identified in the literature include severe coagulopathy, >75% vertebral body collapse, the presence of epidural disease, posterior vertebral body wall fractures, spinal cord compression, and radiculopathy. This emphasizes the importance of involving interventional radiology early in the decision-making process to assess the feasibility and the appropriate type of procedure, and the number of levels that need to be treated to tailor the treatment accordingly. Additional limitations to VAPs include poor performance status, hyperviscosity symptoms, patients with active local or systemic infection, and operating on more than three levels in one session.^41–46^

## Role of Radiotherapy and Radiofrequency Ablation

Stereotactic radiotherapy has been studied as a treatment modality for MBD, secondary to its tumoricidal effect on MM cells, and to block the migration of neoplastic cells by forming an ablation-shell barrier or by embolization of necrotic tumor cells.^47^ The American Society for Radiation Oncology guidelines suggest schemes of a single 8 Gy fraction, 20 Gy in 5 fractions, 24 Gy in 6 fractions, or 30 Gy in 10 fractions, which showed adequate pain relief from painful bone metastasis.^48^ However, radiation doses in excess of 24 Gy pose a higher risk of Grade-I radiation toxicity.^49^ Previous studies using radiotherapy as a stand-alone treatment for MBD demonstrated pain relief ranging from 30%-50%. Although radiotherapy is an excellent non-invasive modality for pain control, it is associated with post-procedural neurological deterioration and continued risk of VCFs.^50–53^

Bludau et al evaluated the role of intraoperative radiotherapy (IORT) during kyphoplasty for vertebral tumors. Although the study had very few patients with myeloma (3/104), combining IORT with kyphoplasty showed excellent long-term local control of the tumor.^54^ At present, in place of VAPs, the current role of stand-alone radiotherapy for treating MBD is minimal. It might be more beneficial in patients with epidural disease, soft tissue plasmacytoma causing cord compression, and neuropathic pain.^50^ The 2018 IMWG consensus statement made recommendations along the same lines for the current role of radiotherapy in MM (Table 3).

**Table 3.** Current indications of radiotherapy in MM as per IMWG^2^

Patients with a soft tissue mass or plasmacytoma that has not resolved with systemic therapy or who cannot receive systemic therapy Palliative approach for poor performance status patients Plasmacytoma associated with severe pain Location of plasmacytoma precluding use of VAPs; e.g., tumor impacting posterior part of the vertebral body close to spinal cord and nerves.

Radiofrequency ablation (RFA) uses a thermal ablation technique which was thought to provide an analgesic effect by the destruction of pain-causing nerves and also reduce the production of cytokines and growth factors by necrosis.^55^ Oregera et al conducted a randomized control trial among 36 patients with MM and MBD to investigate the role of adding RFA to PVP for pain control. One group underwent RFA followed by PVP, and the other underwent PVP alone. Both groups were found to have similar pain relief at 24 hours and 6 weeks post-procedure. It was concluded that adding RFA to PVP provided no extra mid-term pain benefit; however, it increased procedure cost and time.^56^

## Ongoing Studies

Wickstroem et al are conducting a single-blinded, multi-center clinical trial of around 100 patients with MM who had painful vertebral fractures and were randomized to either standard conservative care or standard conservative care with VAP, with the possibility of cross-over within 4 weeks of randomization.^57^ This will be the first nationwide randomized controlled study, and the outcomes will have an impact on the guidelines of patients with MM and painful VCFs.

## Conclusions

VAPs are minimally invasive, safe, and effective for the treatment of VCFs in patients with MM. In addition to treating acute pain associated with VCFs, vertebral augmentation improves anatomic deformity and prevents long-term complications of VCFs, thereby decreasing morbidity and improving QoL. We conclude that, for patients with MM and painful VCFs to obtain the maximum benefit, one of the VAPs should be considered at an early stage, preferably within 4-8 weeks of diagnosis of VCFs if no medical contraindications exist.

### Conflict of Interest

None
